# Hemato-Oncological Diseases as Risk Factor for Recurrence or Metastasis of Pleomorphic Dermal Sarcoma

**DOI:** 10.3389/fonc.2022.873771

**Published:** 2022-04-14

**Authors:** Doris Helbig

**Affiliations:** Department of Dermatology, University Hospital Cologne, Cologne, Germany

**Keywords:** atypical fibroxanthoma (AFX), pleomorphic dermal sarcoma (PDS), immunosuppression, hemato-oncological disease, metastases

## Abstract

**Background:**

Atypical fibroxanthoma (AFX) and pleomorphic dermal sarcoma (PDS) are increasingly common sarcomas of the skin with a genetic UV signature. Immunosuppression is a known risk factor for developing other UV-induced skin cancers such as cutaneous squamous cell carcinoma (cSCC), basal cell carcinoma (BCC), and Merkel cell carcinoma with increased mortality. In case reports or small case series of AFX/PDS patients, immunosuppression has been hypothesized as a risk factor for the development of distant metastases. The aim of the present study was to analyze immunosuppression as a risk factor for AFX/PDS in a large patient cohort.

**Methods:**

A cohort of 164 patients with AFX/PDS (47 AFX and 117 PDS) was collected between 2003 and 2021 and analyzed for clinicopathological data with a special focus on immunosuppression.

**Results:**

Of all patients, 29.9% had any kind of immunosuppression; 6.4% of the AFX and 12.0% of the PDS patients had underlying hemato-oncological diseases. Patients with immunosuppression due to an underlying hemato-oncological disease had a significantly increased risk of progressing to (p = 0.010) and developing distant organ metastases (p = 0.000).

**Conclusions:**

Immunosuppression seems to be a risk factor for developing AFX/PDS with worse clinical outcomes. Therefore, immunosuppression, especially underlying hemato-oncological diseases, should be considered in the treatment and follow-up care of patients with AFX/PDS.

## Introduction

Atypical fibroxanthoma (AFX) and pleomorphic dermal sarcoma (PDS) are defined as rare neoplasms of the skin. Although accurate incidence data do not exist, the incidence is increasing due to demographic changes. Therefore, up to now, they represent the most common sarcomas of the skin (1, 2).

Given the similarities in clinical presentation, histology, and (epi)genetics, AFX and PDS are now considered to be a spectrum of one entity (3–6). They typically occur in chronically light-exposed sites with a non-specific clinical presentation in the form of ulcerated or polypoid tumors (7, 8). Histopathologically, both tumors are very poorly differentiated (“dedifferentiated”) atypical malignant skin neoplasms requiring the exclusion of other poorly differentiated skin tumors such as “dedifferentiated” cutaneous squamous cell carcinoma (cSCC), malignant melanomas, vascular tumors, and other sarcomas, as well as reticulohistiocytomas and atypical fibrous histiocytomas (1, 2, 8–11). Tumor cell morphology includes a variable spectrum of atypical spindle-shaped and epithelioid cells with pleomorphic, vesicular, or hyperchromatic nuclei, as well as atypical multinucleated giant cells and often atypical mitoses, which in the case of AFX remain confined to the dermis and in the case of PDS encompass significant portions of subcutaneous adipose tissue or other deeper structures.

AFX and PDS harbor a UV-induced genetic mutation signature with a very high mutational burden, which is even higher than that of other UV-induced tumors such as cSCC and malignant melanomas (1). The most common genetic alterations include *TP53* loss of function mutations, followed by alterations in *CDKN2A/B* gene (*CDKN2A/B* mutations in 68%, deletions in 71%, and both in 46%) (1, 5, 12).

The local recurrence rate of AFX is less than 5% after complete excision with a lower recurrence rate of patients operated on with microscopically controlled surgery in contrast to surgery with a wide clinical safety margin (13). In PDS, local recurrences have been described in 5%–28% of cases (7, 9, 14, 15), usually occurring within the first 2 years after primary excision. However, a safety margin of 2 cm was associated with a lower local recurrence risk (7). Metastasis rates in PDS range from 8.8% to 20% with an increased risk in very thick primary tumors (7, 9, 14). Metastases are mainly observed in the skin and regional lymph nodes; organ distant metastases are diagnosed in 4% to 10%, most frequently in the lungs. Metastatic cases of AFX published in the literature on the elderly mostly represent PDS according to the current definition (infiltration of subcutis) or were even diagnosed without the use of immunohistochemical markers and are therefore not reliably attributable to AFX/PDS (15–21).

In addition to UV light as a proven etiopathogenetic factor for the development of AFX/PDS, immunosuppression has been recurrently propagated as a risk factor for the development of distant metastases. Nevertheless, in the majority of case reports or case series of metastasized AFX/PDS, the immune status of the patients remains unclear (9, 16, 20, 21).

The importance of the immune system in human skin cancer has been long recognized based primarily upon the increased incidence of skin cancers in organ transplant recipients (OTRs), patients with hemato-oncological diseases, and mechanisms of UV light-mediated immunomodulation.

The present study investigated in a large cohort of patients whether immunosuppression is a risk factor for AFX/PDS in general as well as for advanced-stage disease.

## Materials and Methods

AFX and PDS were selected from the Clinic for Dermatology and Venereology and the archive of Dermatopathology of the University Hospital between 2003 and 2021. AFX and PDS were diagnosed based on the histopathologic criteria described by Fletcher: invasion of the subcutaneous fat or other deeper structures, necrosis, and lymphovascular or perineural invasion were used to distinguish PDS from AFX (22). As part of the diagnostic procedure performed at the time of diagnosis, immunohistochemical staining of at least one cytokeratin (such as p40, pan-cytokeratin, or CK5/6), melanocytic (such as SOX10, S100, melan-A, and HMB45), and vascular markers (such as ERG, CD31, CD34, and podoplanin) as well as desmin had to be performed to exclude potential differential diagnoses such as malignant melanoma, sarcomatoid SCC, leiomyosarcoma, and vascular malignancies.

Mitoses were counted per 10 high-power fields (HPF) and scored according to the FNCLCC Grading System: score 1, 0–9 mitoses per 10 HPF; score 2, 10–19 mitoses per 10 HPF; and score 3, ≥20 mitoses per 10 HPF (23).

Clinicopathological data were retrospectively collected from the medical records of the patients. All tumors had been completely excised. The study protocol conformed to the ethical guidelines of the 1975 Declaration of Helsinki as reflected by the approval of the institution’s human research review committee of the University of Cologne, Germany (registration no. 15-307).

Statistical analyses were performed using the statistical analysis software package IBM SPSS, version 27.0 (Chicago, IL, USA). Pearson’s chi-squared test, t-tests, and univariate hazard ratios were calculated with 95% CIs by use of the Cox proportional hazards model. The significance level was determined at p < 0.05. The Kaplan–Meier curves have been generated for progression-free and overall survival. The significance for the survival curves has been determined using the log-rank test.

## Results and Discussion

A total of 164 patients (47 with AFX and 117 with PDS) were included in our study with a mean follow-up of 25 months (range 0–156 months). The majority of patients were male (89% versus 11%) with a mean age of 79 years at initial diagnosis. The most frequent tumor location was the scalp followed by the trunk and extremities. None of the AFX recurred in contrast to 30 of 117 PDS (25.6%) after complete excision of the primary tumor (see **Table 1**).

**Table 1:** Clinical characteristics of AFX/PDS patients.

Characteristic	AFX	PDS
**Number of patients**	47	117
**Male**	36 (76.6%)	110 (94.0%)
**Female**	11 (23.4%)	7 (6.0%)
**Mean age at initial diagnosis in years ± SD (range)**	78 ± 8 (56–99)	79 ± 8 (43–95)
**Progression-free survival (in months) ± SD (range)**	28 ± 36 (0–156)	18 ± 22 (0–98)
**Overall survival (in months) ± SD (range)**	28 ± 36 (0–156)	24 ± 28 (0–120)
**Primary tumor location**		
**Scalp and neck**	44 (93.6%)	105 (89.7%)
**Trunk**	2 (4.3%)	5 (4.3%)
**Lower extremities**	1 (2.1%)	3 (2.6%)
**Upper extremities**	0	4 (3.4%)
Mitotic number/10 HPF* ± SD (range)		
**Mitotic count**	13.1 ± 6.3 (2–30)	10.1 ± 7.9 (1–50)
**Score 1 **(0–9 mitoses per 10 HPF)	10 (21.3%)	69 (59.0%)
**Score 2** (10–19 mitoses per 10 HPF)	28 (59.6%)	34 (29.1%)
**Score 3** (≥20 mitoses per 10 HPF)	9 (19.1%)	14 (12.0%)
**Progress**	0	30 (25.6%)
**Local recurrence**		16 (13.7%)
**Nodal metastasis**		4 (3.4%)
**Distant (sub)cutaneous metastasis**		5 (4.3%)
**Distant organ metastasis**		5 (4.3%)
**Immunosuppression**		
**No**	32 (68.1%)	82 (70.1%)
**Hemato-oncological disease**	3 (6.4%)	14 (12.0%)
**Others (solid cancer, medical immunosuppression)**	12 (25.5%)	20 (17.1%)
**Unknown**	0	1 (0.9%)

AFX, atypical fibroxanthoma; PDS, pleomorphic dermal sarcoma.

^*^A high-power field (HPF) measures 0.1734 mm^2^ according to the FNCLCC Grading System (23).

Overall, 49 of 164 patients (29.9%) were immunosuppressed; 6.4% of the AFX and 12.0% of the PDS patients had underlying hemato-oncological diseases (see **Table 1**).

Moreover, these immunosuppressed patients with an underlying hemato-oncological disease had a significantly increased risk of progressing to (p = 0.010) and developing distant organ metastases (p = 0.000). In contrast, patients with underlying hemato-oncological diseases were neither more likely to have other solid tumors nor to be on drug immunosuppression. Moreover, the mitotic count of the tumors was similar in patients with underlying hemato-oncological diseases and all other patients (**Table 2**).

**Table 2:** Correlation of immunosuppression due to a hemato-oncological disease and progress or distant organ metastasis.

Characteristic	Patients with immunosuppression due to hemato-oncological disease alone	All other patients	p
**Progress**			
**No**	10 (58.8%)	123 (84.2%)	0.010
**Yes**	7 (41.2%)	23 (15.8%)	
**Distant organ metastasis**			
**No**	14 (82.4%)	144 (98.6%)	0.000
**Yes**	3 (17.6%)	2 (1.4%)	
**Immunosuppression due to solid cancer, medical immunosuppression**			
**No**	17 (85.0%)	117 (80.1%)	0.578
**Yes**	3 (15.0%)	29 (19.9%)	
**Mitotic count**			
**Score 1** (0–9 mitoses per 10 HPF)	10 (58.8%)	68 (46.6%)	0.339
**Score 2–3** (≥10 per 10 HPF)	7 (41.2%)	78 (53.4%)	
**PFS (in months) ± SD (range)**	21 ± 26 (0–75)	21 ± 27 (0–156)	0.992
**OS (in months) ± SD (range)**	21 ± 22 (0–75)	25 ± 31 (0–156)	0.587

HPF, high-power fields; PFS, progression-free survival; OS, overall survival.

Progression-free survival and overall survival were similar in immunosuppressed patients with hemato-oncological diseases as compared to all other patients (see **Table 2**).

This is probably due to the fact that both progression-free survival and overall survival were not achieved in the majority of cases and that many of these elderly patients were lost to follow-up.

Although the exact prevalence of immunosuppression in Germany is unknown, it can be assumed that it is increasing due to greater life expectancy in general and among immunosuppressed adults because of improvements in its medical management as well as new indications for immunosuppressive treatments (24, 25). In a study from the United States, an immunosuppression rate of 2.7% was estimated by self-reports of the adult population.

Up to now, no systematic investigation of immunosuppression as a risk factor for AFX/PDS has been performed. There are only case reports or small case series reporting on immunosuppression in the setting of metastasized AFX/PDS (2, 14–19).

Looking at the most common UV-induced skin cancers, basal cell carcinoma (BCC) and cSCC, the distribution of immunosuppressed versus immunocompetent patients has not been systematically documented. However, a large series in OTRs estimated a 65- to 250-fold increased incidence of cSCC and a 10-fold increased incidence of BCC in renal transplant recipients compared to immunocompetent persons. Moreover, the 3-year disease-specific survival is 56%, with the metastasis rate at 7%, much higher in these patients compared to 0.25% in the general population (26, 27). A first invasive cSCC, often at the base of a field cancerization in chronically UV-damaged skin, typically represents an indicator lesion of an at least 10-fold increased risk for the development of further cSCC in often increasingly shorter time intervals (28). Generally, the cutaneous cancer incidence correlates with the degree and duration of immunosuppression (29).

Regarding other types of immunosuppressed patients, it has been reported that patients with chronic lymphocytic leukemia (CLL) have an 8-fold increased risk for cSCC and BCC with 7 to 14 times increased risk to develop recurrences and/or metastases (30). Hematopoietic cell transplantation recipients have shown a modest risk of skin cancer (31). Age, chronic lymphocytic leukemia, clinically photodamaged skin, and history of cSCC have been defined as independent risk factors for developing cSCC in these patients (32). Interestingly, an azathioprine-specific genetic signature 32 could be detected in both well and poorly differentiated cSCC. Although no therapeutic approach emerges from this study, azathioprine should be avoided in high-risk cSCC patients (33). The risk of Merkel cell carcinoma is also significantly increased in patients with different types of immunosuppression including autoimmune diseases, neoplastic comorbidities, immunosuppression due to hemato-lymphoid disorders such as chronic lymphocytic leukemia/small lymphocytic lymphoma, and immune-modulating drugs or after solid organ transplantation. Moreover, immunosuppression significantly correlated with disease progression in these patients (34–37).

## Conclusions

In our cohort of 164 AFX/PDS patients, around one-third of patients had any kind of immunosuppression, the majority of them due to a hemato-oncological disease, which is significantly more than one would expect in the general population or population of other skin cancers such as cSCC or BCC.

Furthermore, these patients with an underlying hemato-oncological disease progressed significantly more often and developed distant organ metastases significantly more often. These details should be considered in the treatment and follow-up care of these patients.

## Data Availability Statement

The raw data supporting the conclusions of this article will be made available by the author, without undue reservation.

## Author Contributions

The author confirms being the sole contributor of this work and has approved it for publication.

## Conflict of Interest

The author declares that the research was conducted in the absence of any commercial or financial relationships that could be construed as a potential conflict of interest.

## Publisher’s Note

All claims expressed in this article are solely those of the authors and do not necessarily represent those of their affiliated organizations, or those of the publisher, the editors and the reviewers. Any product that may be evaluated in this article, or claim that may be made by its manufacturer, is not guaranteed or endorsed by the publisher.
